# Utility of the monocyte CD64/neutrophil CD64 ratio in autoimmune and autoinflammatory diseases: A retrospective observational study

**DOI:** 10.1016/j.jtauto.2026.100374

**Published:** 2026-05-04

**Authors:** Kazuhiro Itoh, Hiroshi Tsutani, Nozomi Otsuki, Atsushi Kuwata, Chiyo Kiriba, Amane Tanaka, Yasuhiko Mitsuke, Hiromichi Iwasaki, Masamichi Ikawa

**Affiliations:** a Department of Internal Medicine, NHO Awara National Hospital, Awara, Japan; b Department of Community Health Science, Faculty of Medical Sciences, University of Fukui, Fukui, Japan

**Keywords:** Autoinflammatory diseases, Disease flare, Infection, Monocyte-to-neutrophil CD64 ratio, Neutrophil CD64, Systemic autoimmune diseases

## Abstract

Distinguishing superimposed infection from disease flare in systemic autoimmune and autoinflammatory diseases remains challenging, because neutrophil CD64 (nCD64) is sensitive for infection but may have reduced specificity in this setting. We evaluated the monocyte-to-neutrophil CD64 ratio (mCD64/nCD64 ratio) as an adjunctive biomarker in a single-center retrospective study of 408 patients with inflammatory conditions (infection, n = 242; non-infectious inflammation, n = 166). Diagnostic performance of nCD64 and the ratio was assessed by receiver operating characteristic analysis and net reclassification improvement (NRI), including a prespecified subgroup of 86 patients with systemic autoimmune and autoinflammatory diseases to distinguish flares (n = 60) from superimposed infections (n = 26). In the overall cohort, nCD64 showed high accuracy for infection (AUC 0.791), similar to the ratio (AUC 0.764). In the autoimmune/autoinflammatory subgroup, the ratio outperformed nCD64 (AUC 0.926 vs 0.870; p = 0.030) with high sensitivity (96.2%), and improved reclassification (total NRI 0.76; p < 0.001), driven mainly by correct identification of non-infected patients (NRI non-events 0.53). These findings suggest that the mCD64/nCD64 ratio may better distinguish flares from superimposed infections than nCD64 alone in autoimmune/autoinflammatory disease, supporting rule-out decisions when immunosuppressive therapy is being considered.

## Graphical abstract

**Figure:**
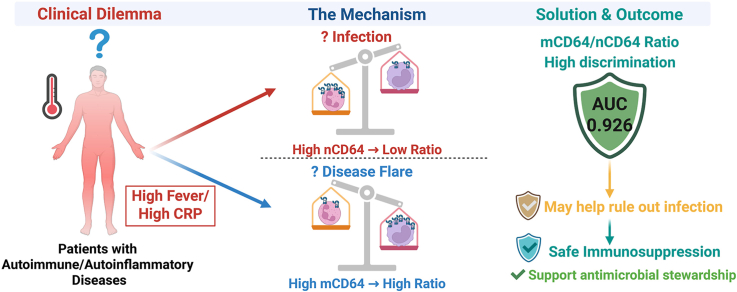

## 1 Introduction

Distinguishing superimposed infections from exacerbations of the underlying condition is one of the most complex issues encountered in the clinical management of systemic autoimmune and autoinflammatory diseases. Patients with these illnesses often exhibit fever and heightened inflammatory markers, but the treatment strategies for the underlying causes are fundamentally contrasting. Misdiagnosis can result in dire consequences: treating a patient with a latent infection with immunosuppressive drugs may trigger sepsis, while delaying necessary immunomodulatory therapy due to an assumed infection might lead to irreparable organ damage. Several studies on systemic lupus erythematosus (SLE) have consistently emphasized the challenge of differentiating infection from disease activity in practical settings [[1], [2], [3], [4], [5]].

Conventional biomarkers, such as C-reactive protein (CRP) and erythrocyte sedimentation rate, are widely used to distinguish between infectious and non-infectious inflammation; however, they lack specificity. Procalcitonin (PCT) has been identified as a specific indicator of bacterial infection; nevertheless, substantial evidence demonstrates that its efficacy is inadequate in localized infections, viral infections, and autoimmune disorders, including SLE [[1], [2], [3], [4], [5], [6], [7]]. Although PCT may provide additional diagnostic value in febrile patients with autoimmune disease, substantial overlap in biomarker levels between infection and flare continues to exist, resulting in a persistent diagnostic gray zone [6].

CD64 (FcγRI) is a high-affinity IgG receptor expressed on neutrophils, monocytes, macrophages, and dendritic cells, and is integral to phagocytosis and cytokine secretion [8]. Neutrophil CD64 (nCD64) expression is minimal in the resting state but is rapidly elevated in response to microbial components, interferon-γ (IFN-γ), and granulocyte colony-stimulating factor (G-CSF) [8,9]. Several studies have identified nCD64 as a sensitive diagnostic biomarker for bacterial infections and sepsis in diverse clinical contexts, including in elderly patients [10], postoperative and critically ill patients [9,11,12], and neonates [13,14]. Otsuki et al. revealed that nCD64 outperformed CRP and leukocyte count in early infection identification in an elderly cohort [10]. Meta-analyses consistently demonstrate that nCD64 is superior to CRP and PCT in terms of diagnostic precision for sepsis [9,11,12].

nCD64 has been demonstrated to differentiate systemic infections from acute inflammatory autoimmune disorders. Allen et al. indicated that nCD64 levels are significantly higher in systemic infections than in flares of rheumatoid arthritis (RA), SLE, or vasculitis, thus affirming its function as a distinguishing biomarker [15]. Nonetheless, its specificity may diminish in acute inflammatory conditions or during corticosteroid therapy [6], suggesting that nCD64 alone cannot address this diagnostic dilemma. In contrast, monocyte CD64 (mCD64) is consistently expressed but significantly increased during macrophage activation. Kikuchi-Taura et al. demonstrated a robust correlation between mCD64 and disease activity in SLE [16], and elevated mCD64 levels have also been reported in adult-onset Still's disease (AOSD), supporting an association with cytokine-driven monocyte/macrophage activation [17]. These data indicate that mCD64 predominantly reflects sterile immune-mediated inflammation rather than infectious stimulation.

The varying expression kinetics suggest that the relative balance of monocyte and neutrophil activation may act as a unique indicator for distinguishing between infectious and non-infectious inflammation. Specifically, elevation of nCD64 signifies infection-induced neutrophil activation, but a selective increase in mCD64 without a corresponding rise in nCD64 may indicate an autoimmune or autoinflammatory flare. The biomarker approach introduced by Kikuchi-Taura et al. supports the prospective diagnostic value of the mCD64/nCD64 ratio [18]. Nonetheless, the combination of mCD64 with nCD64 does not invariably improve diagnostic efficacy; for instance, Li et al. indicated the lack of supplementary benefit of mCD64 in neonatal sepsis [19], underscoring that the ideal expression of these markers may be contingent upon specific contexts.

Therefore, we hypothesized that the mCD64/nCD64 ratio, by integrating contrasting monocyte- and neutrophil-dominant activation patterns, could improve discrimination between infection and non-infectious immune activation. In this retrospective observational study, we aimed to evaluate the diagnostic utility of the mCD64/nCD64 ratio for differentiating infection from non-infectious inflammation overall, with particular focus on distinguishing superimposed infection from disease flare in patients with systemic autoimmune and autoinflammatory diseases.

## 2 Methods

### 2.1 Study design and patient population

This was a single-center, retrospective observational study conducted at a single hospital in Japan. We used a dataset from an ongoing prospective, observational study commenced in 2010, designed to assess CD64 expression in patients with inflammatory conditions (Approval No. 2350). Informed written consent was obtained from all participants upon their enrollment into the prospective registry. For the present retrospective analysis, we extracted data from January 1, 2010, to December 31, 2023. The study protocol was approved by the institutional review board (Approval No. 2401).

We enrolled patients exhibiting clinical symptoms of inflammation, characterized by either fever (axillary temperature ≥37.5 °C) or high CRP (≥0.3 mg/dL). Patients who declined to participate in the study were excluded. Peripheral blood samples were obtained from these patients to assess CD64 expression on leukocytes.

For patients who underwent multiple CD64 measurements during the study period, only the data from the first measurement were included in the analysis to avoid within-patient correlation and to maintain statistical independence.

### 2.2 Sample size determination

The sample size was estimated on the basis of pilot data from our institution collected over the preceding 3 years, including 56 patients with infectious and 80 with non-infectious inflammatory diseases. In this pilot cohort, an mCD64/nCD64 ratio threshold of 15.6 yielded a sensitivity of 61.3% and a specificity of 85.7% for distinguishing infectious from non-infectious inflammatory diseases. Assuming an anticipated sensitivity of 61.3% and a two-sided 95% confidence interval width of ±5%, the required sample size was estimated to be 365 patients. The final study cohort comprised 408 patients, exceeding the prespecified target sample size and thus providing adequate statistical support for the primary analysis.

### 2.3 Flow cytometric analysis of CD64 expression

CD64 expression on neutrophils (nCD64) and monocytes (mCD64) was measured by flow cytometry using residual EDTA-anticoagulated whole blood. Blood samples were obtained at the time of clinical evaluation (i.e., at presentation) before initiation or escalation of antimicrobial or immunosuppressive therapy whenever feasible. Samples were stored at 5 °C and analyzed within 48 h. Whole blood (50 μL) was stained with QuantiBrite CD64 PE/CD45 PerCP (BD Biosciences), followed by red cell lysis/fixation (Optilyse C; Beckman Coulter) and washing. Data were acquired on a FACS Vantage (before Aug 2018) or DxFLEX (after Aug 2018). Neutrophils and monocytes were gated by CD45 and side scatter. CD64 was quantified as molecules per cell using QuantiBrite PE beads, per the manufacturer's instructions.

For patients with febrile neutropenia, CD64 expression was analyzed only if a sufficient number of neutrophils (minimum 100 events in the neutrophil gate) could be acquired to ensure statistical reliability. Herein, all five patients with febrile neutropenia met this quality control criterion.

### 2.4 Longitudinal stability

CD64 was quantified throughout the study using the QuantiBrite PE bead system, which converts fluorescence to molecules per cell (antibodies bound per cell) and supports comparability over time.

### 2.5 Clinical data collection and case definition

Clinical information (age, sex, medical history, medications, and laboratory results including white blood cell count [WBC] and CRP) was extracted from electronic medical records. Patients were classified into infection and non-infection groups using predefined criteria and retrospective adjudication (see below). Briefly, infection was defined by microbiological confirmation or compatible clinical and objective findings, whereas non-infection comprised non-infectious inflammatory conditions in which infection was considered unlikely after review of available investigations.

### 2.6 Diagnosis adjudication

To minimize diagnostic bias, the final diagnosis for each case was retrospectively adjudicated using a structured process. Two reviewers (board-certified rheumatologists and/or infectious disease specialists) independently reviewed each case using predefined criteria and all available clinical information, including microbiological results, imaging findings, and clinical course. Reviewers were blinded to CD64 results (nCD64, mCD64, and the mCD64/nCD64 ratio) during adjudication. Disagreements were resolved by consensus discussion; if consensus could not be reached, a third senior specialist served as an arbitrator.

Cases were classified as infection or non-infectious inflammatory disease activity (flare). Infection was adjudicated when there was microbiological confirmation from a sterile site or clinically relevant specimen, or when clinical and radiological findings were strongly compatible with bacterial infection and no alternative non-infectious explanation was identified. Non-infectious flare was adjudicated when infection was considered unlikely after review of available investigations and the presentation was consistent with active autoimmune/autoinflammatory disease supported by disease activity assessments where available. Response to antimicrobial therapy or immunosuppressive escalation was considered supportive information, but not a defining criterion for classification.

### 2.7 Statistical analysis

Continuous variables are presented as median and interquartile range (IQR). The Mann–Whitney *U* test was employed to assess the differences between the two groups. To evaluate the diagnostic efficacy of the biomarkers (WBC, CRP, nCD64, mCD64, and mCD64/nCD64 ratio), receiver operating characteristic (ROC) curves were generated, and the area under the curve (AUC) was determined. The optimal cutoff values were established using the Youden index. To assess the additional diagnostic utility of the mCD64/nCD64 ratio compared to traditional markers or nCD64 alone, we computed the continuous net reclassification improvement (NRI). Continuous NRI was calculated using infection as the event. We additionally performed decision curve analysis to evaluate clinical utility; predicted probabilities were obtained from logistic regression models using nCD64 or the mCD64/nCD64 ratio, and net benefit was calculated across a range of threshold probabilities. Statistical analyses were conducted using EZR version 1.68 (Saitama Medical Center, Jichi Medical University, Japan) [20] and GraphPad Prism version 10.6.1 (GraphPad Software, Boston, MA, USA) for general statistics and ROC analysis. The NRI and decision curve analysis computation were performed in R statistical software (The R Foundation for Statistical Computing, Vienna, Austria). A p-value <0.05 was deemed statistically significant. As an exploratory analysis prompted by reviewer comments, cases in the overall cohort were further classified as bacterial infection, viral infection, or non-infectious inflammation after exclusion of fungal infection, febrile neutropenia, and scabies because of their heterogeneity and small numbers. Biomarker distributions were compared across these groups using the Kruskal–Wallis test followed by Dunn's multiple comparison test. In addition, a sensitivity analysis was performed comparing bacterial infection alone with non-infectious inflammation.

### 2.8 Patient and public involvement

Patients were not involved in the design, conduct, reporting, or dissemination of this research.

Additional analyses are provided in the Supplementary Appendix (Figures S1–S7, Tables S1–S2, and Text S1).

## 3 Results

### 3.1 Patient characteristics

**Fig. 1:**
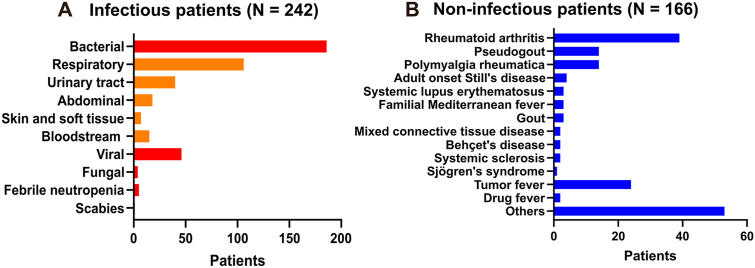
**Study profile and distribution of final diagnoses in the overall cohort and in the autoimmune/autoinflammatory disease subgroup.** The study included 408 patients with inflammatory conditions who underwent CD64 measurement and were classified as having infection (n = 242) or non-infectious inflammation (n = 166). The figure summarizes patient selection and the final diagnostic categories in the overall cohort, including bacterial, viral, fungal, and other infections, as well as non-infectious inflammatory conditions. The autoimmune/autoinflammatory disease subgroup comprised 86 patients, including 26 with superimposed infection and 60 with disease flare. Representative non-infectious diagnoses included systemic lupus erythematosus, rheumatoid arthritis, adult-onset Still's disease, and familial Mediterranean fever.

A total of 408 patients were included (Fig. 1), comprising 242 patients with infection and 166 with non-infectious inflammation. Baseline characteristics are summarized in Table 1. The cohort was predominantly elderly (median age 82 years [IQR 70–89]), and 154 patients (37.8%) were male. Overall, the two groups were broadly comparable, although patients in the infection group were slightly older and glucocorticoid use was more frequent in the non-infection group.

**Table 1:** Baseline characteristics and laboratory findings of patients with infection and non-infectious inflammation in the overall cohort.

Variable	Total (n = 408)	Infection Group (n = 242)	Non-infection Group (n = 166)	p-value^b^
Demographics				
Age, years, median (IQR)	82 (70–89)	82.5 (74–90)	80 (68–87)	0.010
Male sex, n (%)^a^	154 (37.8)	91 (37.6)	63 (38.2)	0.907
Medication				
Glucocorticoid use, n (%)	17 (4.2)	2 (0.8)	15 (9.0)	<0.001
Laboratory Findings				
WBC,/μL, median (IQR)	7200 (5375–9525)	7250 (5300–9775)	7100 (5525–9375)	0.691
CRP, mg/dL, median (IQR)	4.2 (1.3–8.2)	5.4 (1.7–9.5)	2.6 (0.7–6.9)	<0.001
nCD64, molecules/cell, median (IQR)	2082 (1287–4135)	3095 (1815–5314)	1350 (960–1932)	<0.001
mCD64, molecules/cell, median (IQR)	26,795 (21,410–34,434)	29,201 (23,264–39,257)	25,064 (19,440–29,954)	<0.001
mCD64/nCD64 Ratio, median (IQR)	12.2 (7.7–18.5)	9.3 (6.1–14.1)	17.0 (12.2–23.4)	<0.001
Diagnosis, n (%)				
Infectious Diseases	242 (59.3)	242 (100)	0 (0)	-
Bacterial infection	186 (45.6)	186 (76.9)	-	
Viral infection	46 (11.3)	46 (19.0)	-	
Others	10 (2.5)	10 (4.1)	-	
Non-infectious Diseases	166 (40.7)	0 (0)	166 (100)	-
Rheumatoid arthritis	39 (9.6)	-	39 (23.5)	
Tumor fever	24 (5.9)	-	24 (14.5)	
Pseudogout	14 (3.4)	-	14 (8.4)	
Polymyalgia rheumatica	14 (3.4)	-	14 (8.4)	
Others	75 (18.4)	-	75 (45.2)	

Note.

Data are presented as median (interquartile range) or n (%), as appropriate. The infection group included bacterial, viral, fungal, and other infections. “Others” denotes infections not classified as bacterial, viral, or fungal, including febrile neutropenia and scabies. Laboratory variables were measured at the time of CD64 assessment. CRP, C-reactive protein; mCD64, monocyte CD64; nCD64, neutrophil CD64; WBC, white blood cell count.

a Male sex was unknown for 1 patient.

b P values were calculated using the Mann–Whitney *U* test for continuous variables and the χ^2^ test or Fisher's exact test for categorical variables, as appropriate.

### 3.2 Comparison of biomarker levels between groups

We compared inflammatory biomarker levels between the infection and non-infection groups (Supplementary Fig. S1A–E). Among the individual biomarkers, nCD64 showed the clearest increase in the infection group. Conventional inflammatory markers, including CRP and WBC count, were also significantly higher in infection. In contrast, the mCD64/nCD64 ratio showed an inverse pattern and was significantly higher in the non-infection group, suggesting that this index may better reflect non-infectious immune activation than infection.

### 3.3 Diagnostic performance of biomarkers in the overall cohort

The diagnostic performance of each biomarker in the overall cohort is summarized in Table 2 nCD64 showed the highest diagnostic accuracy among the individual biomarkers for differentiating infection from non-infectious inflammation. By contrast, conventional markers had limited discriminatory value. In the overall cohort, the mCD64/nCD64 ratio did not outperform nCD64 alone, indicating that nCD64 remains the more useful single marker for general infection screening (Supplementary Fig. S2).

**Table 2:** Diagnostic performance of white blood cell count, C-reactive protein, monocyte CD64, neutrophil CD64, and the mCD64/nCD64 ratio for differentiating infection from non-infectious inflammation and for distinguishing superimposed infection from disease flare in autoimmune/autoinflammatory diseases.

Cohort/Biomarker	Cutoff value	AUC (95% CI)	Sensitivity (%)	Specificity (%)	LR+	LR-
Overall cohort (n = 408)						
nCD64 (molecules/cell)	1948	0.791 (0.747–0.836)	72.7	75.9	3.02	0.36
mCD64/nCD64 Ratio	13.5	0.764 (0.717–0.810)	73.1	71.7	2.58	0.38
mCD64 (molecules/cell)	30,895	0.645 (0.592–0.698)	45.5	79.5	2.22	0.69
CRP (mg/dL)	4.2	0.613 (0.556–0.669)	58.7	62.7	1.57	0.66
WBC (/μL)	11,700	0.512 (0.455–0.568)	17.4	95.2	3.63	0.87
Subgroup (n = 86)‡						
mCD64/nCD64 Ratio	12.7	0.926 (0.867–0.984)	96.2	76.7	4.13	0.05
nCD64 (molecules/cell)	1825	0.870 (0.792–0.948)	84.6	76.7	3.63	0.20
CRP (mg/dL)	1.0	0.675 (0.543–0.807)	84.6	50.0	1.69	0.31
WBC (/μL)	7000	0.619 (0.478–0.759)	65.4	61.7	1.71	0.56
mCD64 (molecules/cell)	41,266	0.528 (0.388–0.668)	23.1	93.3	3.45	0.82

Note.

Diagnostic performance was evaluated by receiver operating characteristic analysis. The table shows the area under the curve (AUC), 95% confidence interval (CI), optimal cutoff value, sensitivity, specificity, positive likelihood ratio (LR+), and negative likelihood ratio (LR−) for each biomarker. In the overall cohort, biomarkers were assessed for differentiating infection from non-infectious inflammation. In the subgroup analysis, biomarkers were assessed for distinguishing superimposed infection from disease flare in patients with underlying autoimmune/autoinflammatory diseases. AUC, area under the curve; CI, confidence interval; CRP, C-reactive protein; LR+, positive likelihood ratio; LR−, negative likelihood ratio; mCD64, monocyte CD64; nCD64, neutrophil CD64; WBC, white blood cell count.

### 3.4 Subgroup analysis: differentiating infection from autoimmune and autoinflammatory disease flare

We next examined the subgroup of 86 patients with systemic autoimmune and autoinflammatory diseases, including 26 with superimposed infection and 60 with disease flare. Baseline characteristics are shown in Supplementary Table S1. Age, sex, underlying diseases, and glucocorticoid use did not differ significantly between the two groups.

**Fig. 2:**
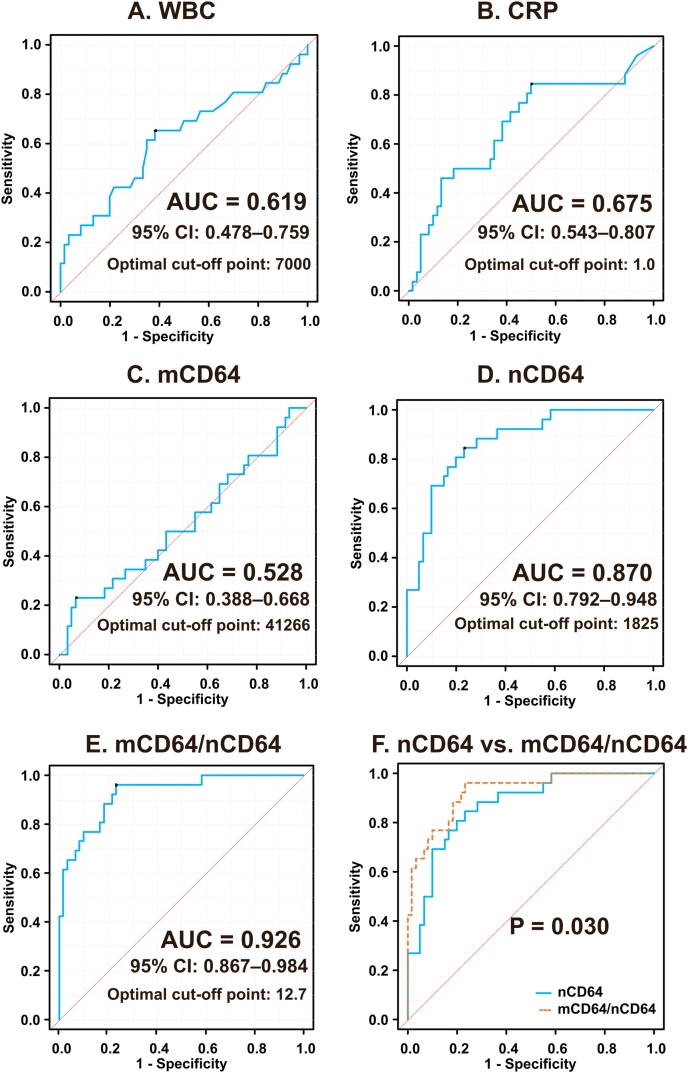
**Receiver operating characteristic (ROC) curves of inflammatory biomarkers for differentiating infection from non-infectious inflammation in the overall cohort and for distinguishing superimposed infection from disease flare in the autoimmune/autoinflammatory disease subgroup.** ROC curves are shown for white blood cell count (WBC), C-reactive protein (CRP), monocyte CD64 (mCD64), neutrophil CD64 (nCD64), and the mCD64/nCD64 ratio. (A) In the overall cohort (n = 408), nCD64 showed the highest diagnostic accuracy for differentiating infection from non-infectious inflammation. (B) In patients with autoimmune/autoinflammatory diseases (n = 86), the mCD64/nCD64 ratio outperformed nCD64 alone for distinguishing superimposed infection (n = 26) from disease flare (n = 60), supporting its potential utility as a rule-out marker for infection in this clinical context. AUC values with 95% CIs are presented in the figure.

Biomarker comparisons are shown in Supplementary Fig. S1F–J and Supplementary Table S1. CRP and nCD64 were significantly higher in the infection group, whereas mCD64 did not differ significantly between infection and flare. In contrast, the mCD64/nCD64 ratio was significantly higher in the flare group than in the infection group. Diagnostic performance analysis showed that the ratio achieved the highest accuracy in this subgroup (AUC 0.926, 95% CI 0.867–0.984), and direct ROC comparison confirmed that it significantly outperformed nCD64 alone (0.926 vs 0.870, p = 0.030; Fig. 2). These findings indicate that incorporating the monocyte–neutrophil activation balance improves discrimination between flare and superimposed infection in this clinically challenging setting.

### 3.5 Reclassification analysis

To further assess clinical utility beyond AUC comparison, we performed continuous NRI analysis using infection as the event (Table 3). The mCD64/nCD64 ratio significantly improved classification compared with nCD64 alone in the autoimmune/autoinflammatory subgroup. This improvement was driven mainly by better identification of non-infected patients with disease flare. Among the 60 flare cases, the ratio reduced false-positive classification of infection in 46 cases (76.7%), corresponding to an NRI for non-events of 0.53 (p < 0.001). These results support the particular value of the ratio as a rule-out marker for infection in patients with autoimmune/autoinflammatory disease. Decision curve analysis also suggested greater net benefit for the ratio than for nCD64 alone across clinically relevant threshold probabilities (Supplementary Fig. S4).

**Table 3:** Continuous net reclassification improvement analysis comparing neutrophil CD64 alone with the mCD64/nCD64 ratio in patients with autoimmune/autoinflammatory diseases.

Statistic	Value (95% CI)	p-value	Improved (n)	Worsened (n)
Total NRI	0.76 (0.294–1.192)	<0.001		
NRI for events (Infection)	0.23 (−0.154–0.600)	0.233	16 (61.5%)	10 (38.5%)
NRI for non-events (Non-Infection)	0.53 (0.316–0.744)	<0.001	46 (76.7%)	14 (23.3%)

Note.

Continuous net reclassification improvement (NRI) was calculated using infection as the event. Events refer to patients with superimposed infection (n = 26), and non-events refer to patients with disease flare (n = 60). Positive NRI values indicate improved classification by the mCD64/nCD64 ratio compared with nCD64 alone. NRI, net reclassification improvement; mCD64, monocyte CD64; nCD64, neutrophil CD64.

### 3.6 Analysis of the non-autoimmune/autoinflammatory subgroup

We then examined the remaining 322 patients without systemic autoimmune or autoinflammatory diseases to assess whether the advantage of the ratio was disease-context dependent. In this subgroup, nCD64 performed better than the mCD64/nCD64 ratio, with a significantly higher AUC (0.771 vs 0.737, p = 0.043; Supplementary Fig. S3). Reclassification analysis also favored nCD64 alone (total NRI = 0.45, p < 0.001; Supplementary Table S2), mainly because of better specificity in non-events. These findings suggest that the added value of the ratio is specific to patients with autoimmune/autoinflammatory backgrounds and is not generalizable to the broader population with inflammatory conditions.

To further contextualize these findings, we performed an exploratory analysis according to infection subtype.

### 3.7 Exploratory analyses by infection subtype

To examine the potential influence of viral infection, we performed an exploratory analysis in the overall cohort after classifying cases as bacterial infection (n = 186), viral infection (n = 46), or non-infectious inflammation (n = 166), excluding fungal infection, febrile neutropenia, and scabies because of their heterogeneity and small numbers. Bacterial infection showed the clearest inflammatory profile, with higher WBC, CRP, and nCD64 levels and a lower mCD64/nCD64 ratio than the other two groups (Supplementary Fig. S6). By contrast, viral infection and non-infectious inflammation overlapped substantially for nCD64 and the mCD64/nCD64 ratio, although mCD64 remained higher in viral infection than in non-infectious inflammation.

In a sensitivity analysis restricted to bacterial infection versus non-infectious inflammation, nCD64 and the mCD64/nCD64 ratio showed similar overall diagnostic performance, although nCD64 retained slightly higher specificity (Supplementary Fig. S7).

## 4 Discussion

This study assessed the diagnostic value of the mCD64/nCD64 ratio in distinguishing between infectious and non-infectious inflammation, especially in patients with systemic autoimmune and autoinflammatory diseases. Although nCD64 alone was an excellent indicator of infection in the total population, subgroup analysis demonstrated that the mCD64/nCD64 ratio considerably outperformed nCD64 in patients with underlying systemic autoimmune and autoinflammatory diseases. Moreover, reclassification analysis (NRI) indicated that the ratio is especially effective in detecting non-infectious disease flares, thereby mitigating the risk of erroneously classifying these patients as having infections. In decision curve analysis, the ratio also showed higher net benefit than nCD64 across a range of threshold probabilities, supporting its potential utility in clinical decision-making (Supplementary Fig. S4). These findings suggest that the mCD64/nCD64 ratio serves as a clinically accessible readout of myeloid activation balance, integrating relative monocyte- and neutrophil-lineage responses in inflammatory disease.

nCD64 showed significant diagnostic accuracy (AUC 0.791) in our total cohort (N = 408), corroborating previous findings that it is a more effective marker for bacterial infection than CRP and WBC [11,12,21]. The expression of nCD64 is rapidly enhanced by pro-inflammatory cytokines, including IFN-γ and G-CSF, during bacterial invasion, acting as a highly sensitive indicator of sepsis. In the general population, where bacterial infection is the primary source of inflammation, a significant increase in nCD64 masks alterations in mCD64 expression, enhancing the diagnostic ability of nCD64 alone.

The diagnostic scenario becomes complex in patients with systemic autoimmune and autoinflammatory diseases. Although nCD64 is a sensitive marker of infection, its specificity may be reduced in autoimmune/autoinflammatory conditions in which innate immune activation also involves neutrophil-mediated inflammatory pathways, including NETosis [22]. In conditions such as SLE and AOSD, aberrant macrophage activation is a central pathogenic feature. Monocytes and macrophages play crucial roles in the pathogenesis of these diseases through the production of pro-inflammatory cytokines and antigen presentation [23,24]. Specifically, in autoinflammatory conditions, such as AOSD, activation of the NLRP3 inflammasome in monocytes leads to the excessive release of IL-1β and IL-18, driving a distinct inflammatory cascade [25]. Previous reports have indicated that mCD64 is constitutively expressed but significantly upregulated in the active phases of these diseases, reflecting this monocyte-lineage activation [17,26]. Our subgroup data revealed that mCD64 levels were robustly elevated in both the infection and flare groups, with no significant differences observed. This confirms that monocyte activation is a shared feature of both pathologies. However, the magnitude of nCD64 upregulation in bacterial infection was disproportionately higher, overshadowing the monocyte activation and resulting in a lower mCD64/nCD64 ratio. In contrast, nCD64 elevation was generally mild in systemic autoimmune and autoinflammatory disease flares compared with that in severe bacterial infections, whereas mCD64 remained robustly elevated in these disease flares, comparable to the levels observed in infections. Consequently, the balance shifted toward a higher ratio in non-infectious flares. Overall, the mCD64/nCD64 ratio reflects relative monocyte-dominant versus neutrophil-dominant activation, helping distinguish flares from acute bacterial infections.

While the mCD64/nCD64 ratio performed best in patients with systemic autoimmune and autoinflammatory diseases, nCD64 alone provided better reclassification in patients without these conditions. This finding is consistent with prior reports showing nCD64 as a robust infection marker in general populations [[10], [11], [12], [13]]. Taken together, these results support a stratified strategy: use the mCD64/nCD64 ratio when systemic autoimmune or autoinflammatory disease is present, and use nCD64 alone for general infection screening.

The most clinically relevant finding of this study is the improvement in diagnostic certainty afforded by the ratio, as demonstrated by the NRI analysis. Among patients with systemic autoimmune and autoinflammatory diseases, the overall NRI was 0.76 (p < 0.001), predominantly influenced by the improvement in the non-infection cohort (NRI non-events = 0.53). This implies that approximately 53% of patients with disease flares, who would have been misclassified as having infections based on nCD64 alone, were correctly reclassified as non-infected using the ratio. Physicians encounter a therapeutic dilemma: the administration of immunosuppressive therapy to patients with latent infection may trigger sepsis or septic shock, while refraining from treatment for a disease flare in favor of unnecessary antibiotics can lead to irreversible organ damage and promote antimicrobial resistance [11,12]. Our data indicated that a high mCD64/nCD64 ratio may serve as a useful “rule-out” diagnostic indicator for infection. Accordingly, we propose a stratified diagnostic approach for clinical use (Supplementary Fig. S5 and Text S1), in which nCD64 is used for general infection screening and the mCD64/nCD64 ratio is prioritized when systemic autoimmune or autoinflammatory disease is present. The subgroup analysis demonstrated a high sensitivity of 96.2% and low negative likelihood ratio (0.05), thereby ruling out infection and enabling clinicians to confidently commence or escalate immunosuppressive therapy. The accurate reclassification of 76.7% of patients in the non-infection group in this subgroup, whose classification may have been unclear based on nCD64 only, indicates that this ratio could significantly contribute to antimicrobial stewardship in this high-risk population.

In the overall cohort, we observed a significantly higher prevalence of glucocorticoid use in the non-infection group than in the infection group (9.0% vs. 0.8%, p < 0.001). Glucocorticoids are known to suppress inflammatory responses and can affect CD64 expression levels [6,16], raising concerns about potential confounding. However, crucially, in our subgroup analysis of patients with systemic autoimmune and autoinflammatory diseases, there was no statistically significant difference in glucocorticoid use between the infection and disease flare groups (0% vs. 13.3%, p = 0.099). This suggests that the superior diagnostic performance of the mCD64/nCD64 ratio in this specific population is not an artifact of treatment but reflects the fundamental pathological difference between monocyte-driven autoimmunity and neutrophil-driven infection.

Furthermore, the relatively low rate of glucocorticoid use in the non-infection group (9.0%) likely reflects our study design, in which only the first CD64 measurement per patient was analyzed. As a result, the cohort may have included a considerable number of individuals at the time of initial presentation, before immunosuppressive therapy had been started. In this context, the diagnostic performance of the mCD64/nCD64 ratio appears applicable not only to disease monitoring but also to the initial evaluation of untreated patients. This interpretation is consistent with previous studies reporting marked upregulation of mCD64 during active phases of AOSD and SLE, particularly in treatment-naïve cases [8,17].

These additional analyses help refine the clinical interpretation of our findings. Viral infection, which accounted for a substantial proportion of infection cases in the overall cohort, showed biomarker distributions distinct from bacterial infection but closer to non-infectious inflammation, particularly for nCD64 and the mCD64/nCD64 ratio. This suggests that the discriminatory strength of these markers is driven mainly by their ability to identify bacterial infection rather than to separate viral infection from immune-mediated inflammatory activity. In this context, the mCD64/nCD64 ratio may be best interpreted as a marker of myeloid activation balance that helps rule out bacterial infection in inflammatory presentations, rather than as a universal discriminator of all infectious etiologies. Other host-response leukocyte markers relevant to viral infection and immune activation have also been investigated, including flow-cytometry–based approaches incorporating CD64 and CD169 [27]. However, such comparator markers were not routinely available in our cohort, and their incremental value relative to the mCD64/nCD64 ratio remains to be determined.

This study has limitations. First, it was a single-center retrospective study, which may limit generalizability to other settings. Second, although the overall sample size was substantial (n = 408), the number of infection cases in the systemic autoimmune and autoinflammatory disease subgroup was relatively small (n = 26); therefore, estimates of diagnostic performance in this subgroup should be interpreted cautiously. Third, we analyzed systemic autoimmune and autoinflammatory diseases as a combined category, despite heterogeneity in underlying cytokine biology across conditions (e.g., SLE, rheumatoid arthritis, and adult-onset Still's disease). Fourth, viral infection represented a clinically important subgroup in the overall cohort, but the number of viral infection cases within the systemic autoimmune and autoinflammatory disease subgroup was very limited. In that subgroup, viral infection comprised only three cases (one COVID-19 case and two cases of acute pharyngitis), precluding robust subgroup-specific inference. Our exploratory analysis in the overall cohort suggested that viral infection showed biomarker distributions closer to non-infectious inflammation than to bacterial infection, particularly for nCD64 and the mCD64/nCD64 ratio. Therefore, the present study was not designed to establish a viral-specific diagnostic signature in autoimmune/autoinflammatory disease, and this question should be addressed in future studies enriched for viral infection. Potentially relevant comparator biomarkers, including PCT and other host-response leukocyte markers, were not routinely available in this cohort, precluding direct comparison of the mCD64/nCD64 ratio with these clinically relevant alternative markers.

In conclusion, although nCD64 is a dependable indicator of infection in the general population, the mCD64/nCD64 ratio provides enhanced diagnostic precision in individuals with preexisting autoimmune and autoinflammatory conditions. The ratio accurately differentiates disease flares from concurrent infections by representing the equilibrium between monocyte and neutrophil activity. This index may support treatment decisions, antimicrobial stewardship, and outcomes in this complex patient population.

## Data access and verification

Kazuhiro Itoh and Hiroshi Tsutani directly accessed and verified the underlying data reported in this study. Masamichi Ikawa had full access to all the data and had final responsibility for the decision to submit for publication.

## Ethics statement

This study was approved by the Institutional Review Board of NHO Awara National Hospital (approval number: 2401). Where applicable, data were derived from an ongoing prospective observational registry (approval number: 2350) with written informed consent obtained at enrolment.

## Clinical trial number

Not applicable.

## Consent to publish declaration

Not applicable.

## Generative AI statement

During the preparation of this work, the authors used generative AI (ChatGPT, OpenAI) for language editing and to improve readability. The authors reviewed and edited the content and take full responsibility for the final manuscript.

## Funding

This work was supported by a Grant-in-Aid for Clinical Research from the National Hospital Organization (Grant Number R6-NHO(PI)-12). The funder had no role in study design, data collection, data analysis, data interpretation, writing of the report, or the decision to submit for publication.

## CRediT authorship contribution statement

**Kazuhiro Itoh:** Conceptualization, Data curation, Formal analysis, Funding acquisition, Investigation, Methodology, Project administration, Resources, Software, Validation, Visualization, Writing – original draft, Writing – review & editing. **Hiroshi Tsutani:** Conceptualization, Data curation, Methodology, Writing – original draft, Writing – review & editing. **Nozomi Otsuki:** Conceptualization, Data curation, Methodology, Writing – original draft, Writing – review & editing. **Atsushi Kuwata:** Conceptualization, Data curation, Methodology, Writing – review & editing. **Chiyo Kiriba:** Conceptualization, Data curation, Methodology, Writing – review & editing. **Amane Tanaka:** Conceptualization, Data curation, Methodology, Writing – review & editing. **Yasuhiko Mitsuke:** Conceptualization, Data curation, Methodology, Writing – original draft, Writing – review & editing. **Hiromichi Iwasaki:** Conceptualization, Methodology, Supervision, Writing – original draft, Writing – review & editing. **Masamichi Ikawa:** Conceptualization, Methodology, Supervision, Writing – original draft, Writing – review & editing.

## Declaration of competing interest

The authors declare the following financial interests/personal relationships which may be considered as potential competing interests: Kazuhiro Itoh reports financial support was provided by National Hospital Organization. If there are other authors, they declare that they have no known competing financial interests or personal relationships that could have appeared to influence the work reported in this paper.

## Data Availability

Data will be made available on request.
