# Supplementary material for: Utility of the monocyte CD64/neutrophil CD64 ratio in autoimmune and autoinflammatory diseases: A retrospective observational study
Source: J Transl Autoimmun. 2026 May 4;12:100374. doi: 10.1016/j.jtauto.2026.100374 (PMC13186010; doi:10.1016/j.jtauto.2026.100374)
Supplement: Multimedia component 1 [file mmc1.docx]

**Supplementary Appendix
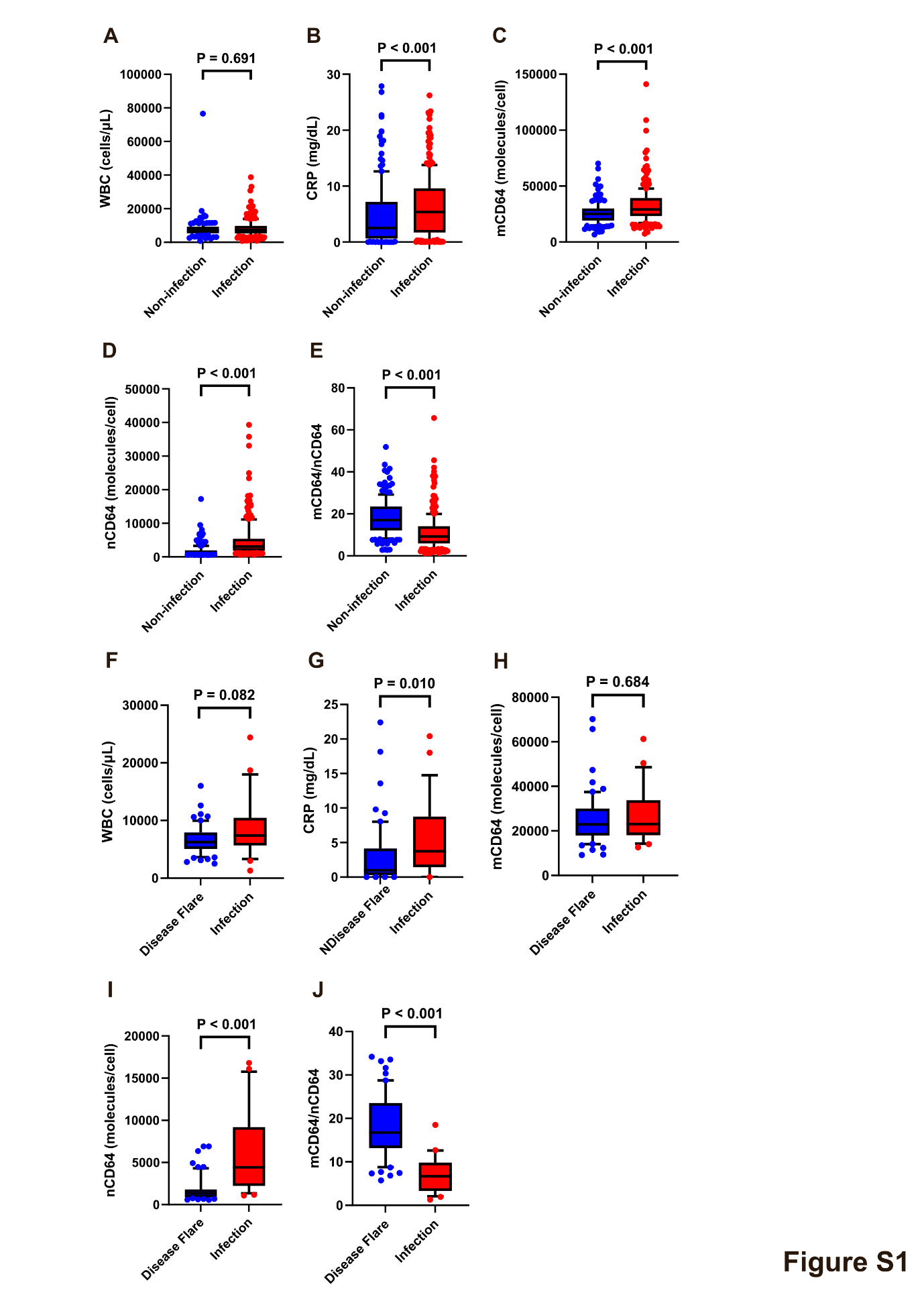
**

**Figure S1. Comparative analysis of inflammatory biomarkers between infection and non-infection groups.** Box plots illustrating (A) white blood cell count (WBC), (B) C-reactive protein (CRP), (C) monocyte CD64 (mCD64), (D) neutrophil CD64 (nCD64), and (E) the mCD64/nCD64 ratio. Although nCD64, mCD64, CRP, and WBC levels were significantly elevated in the infection group, the mCD64/nCD64 ratio exhibited an inverse trend, being significantly higher in the non-infection group (Mann–Whitney U test, p < 0.001). (F–J) Subgroup of patients with autoimmune/autoinflammatory diseases (n = 86): box plots comparing superimposed infection (n = 26) vs. disease flare (n = 60). (F) WBC showed no significant difference. (G) CRP and (I) nCD64 were significantly higher in the infection group. (H) mCD64 showed no significant difference between the groups (p = 0.684), indicating elevated monocyte activation in both conditions. (J) The mCD64/nCD64 ratio was significantly higher in the flare group (p < 0.001), clearly distinguishing it from the infection group. The center line denotes the median, the box indicates the interquartile range (25th–75th percentiles), and the whiskers indicate the 10th–90th percentiles; values outside the whiskers are plotted as individual points.


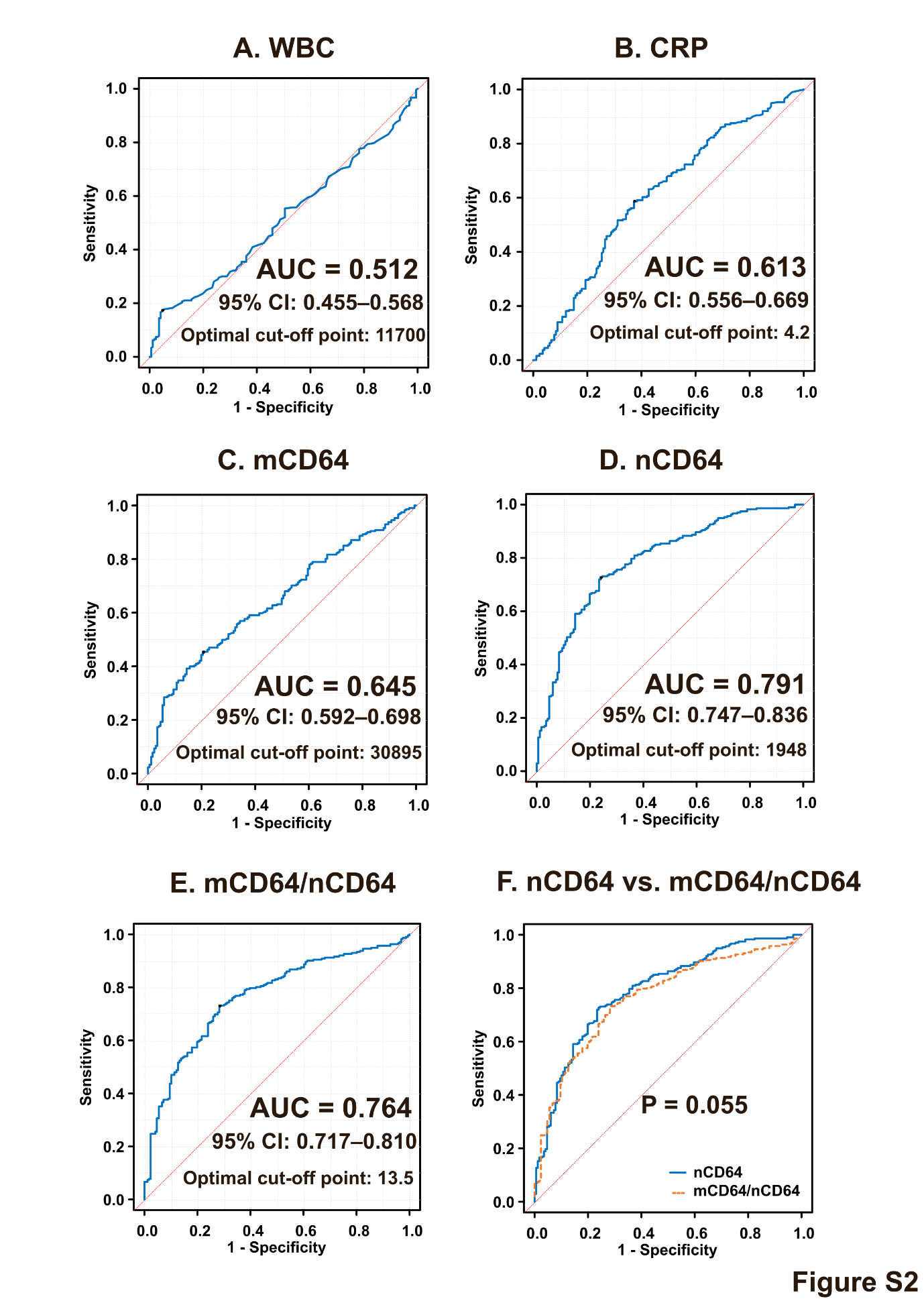


**Figure S2. Diagnostic performance of individual biomarkers in the total cohort (n = 408).** Receiver operating characteristic (ROC) curves for distinguishing infection from non-infectious inflammation using (A) white blood cell count (WBC), (B) C-reactive protein (CRP), (C) monocyte CD64 (mCD64), (D) neutrophil CD64 (nCD64), and (E) the mCD64/nCD64 ratio. nCD64 exhibited the highest area under the curve (AUC) compared to the individual markers. AUC values: nCD64 (0.791), mCD64/nCD64 ratio (0.764), mCD64 (0.645), CRP (0.613), WBC (0.512). (F) Comparative analysis of ROC curves for nCD64 and the mCD64/nCD64 ratio. The ROC curves for nCD64 (orange line) and the mCD64/nCD64 ratio (blue line) are compared. AUC values: nCD64 (0.791) compared to mCD64/nCD64 ratio (0.764). The two AUCs exhibited no statistically significant difference (p = 0.055).

**
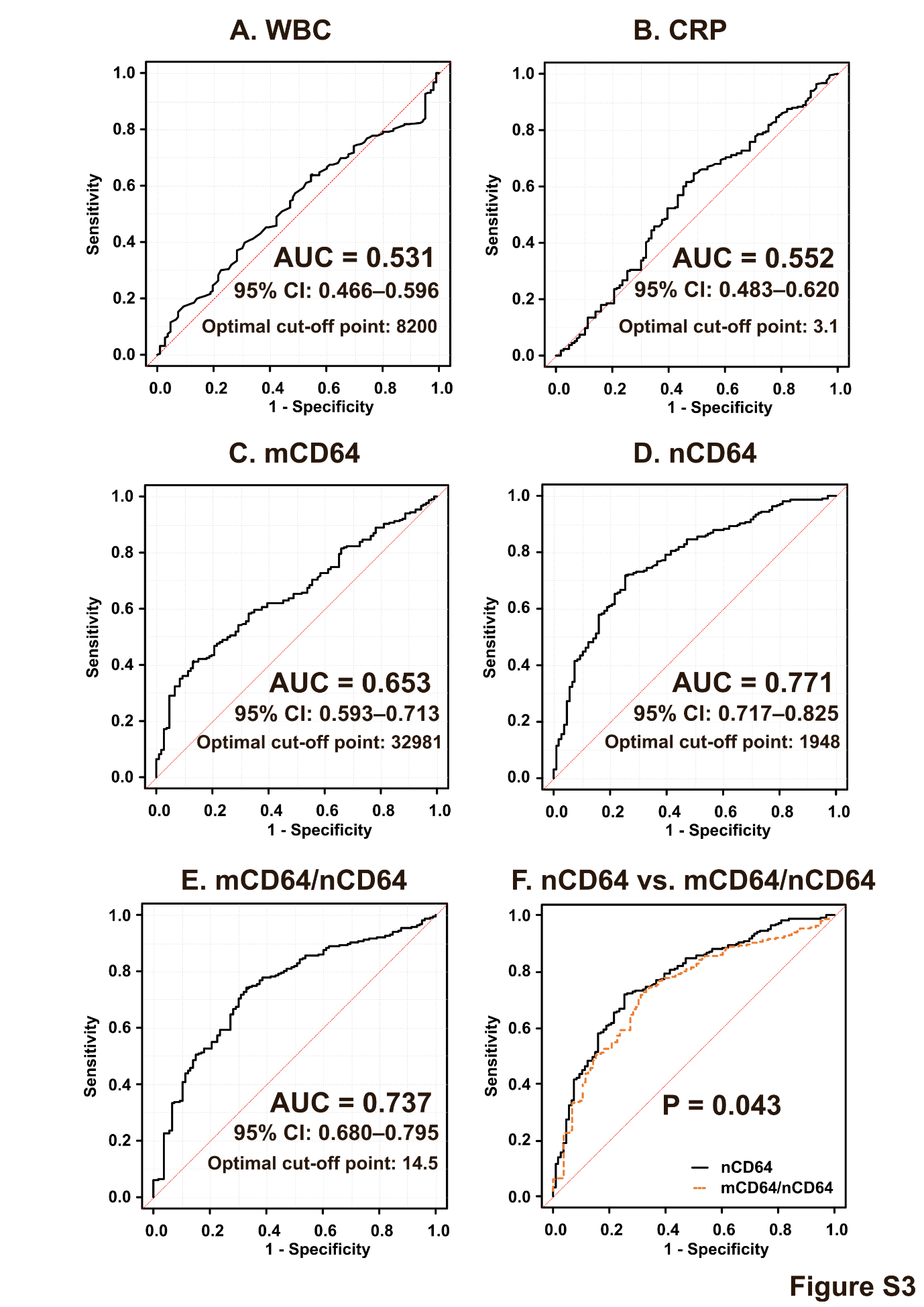
**

**Figure S3. Receiver operating characteristic (ROC) curves of biomarkers in the subgroup of patients without autoimmune or autoinflammatory diseases (n = 322).** Diagnostic performance of (A) white blood cell count (WBC), (B) C-reactive protein (CRP), (C) monocyte CD64 (mCD64), (D) neutrophil CD64 (nCD64), and (E) the mCD64/nCD64 ratio in distinguishing infection from non-infectious inflammation. Consistent with general population studies, nCD64 demonstrated the highest area under the curve (AUC) of 0.771 among the tested biomarkers. (F) Statistical comparison of ROC curves for nCD64 (black solid line) and the mCD64/nCD64 ratio (orange dashed line). In contrast to that in the subgroup of patients with systemic autoimmune and autoinflammatory diseases, the AUC of nCD64 (0.771) was significantly higher than that of the mCD64/nCD64 ratio (0.737) in this population (p = 0.043). This confirms that the superior utility of the ratio is specific to patients with systemic autoimmune and autoinflammatory diseases.


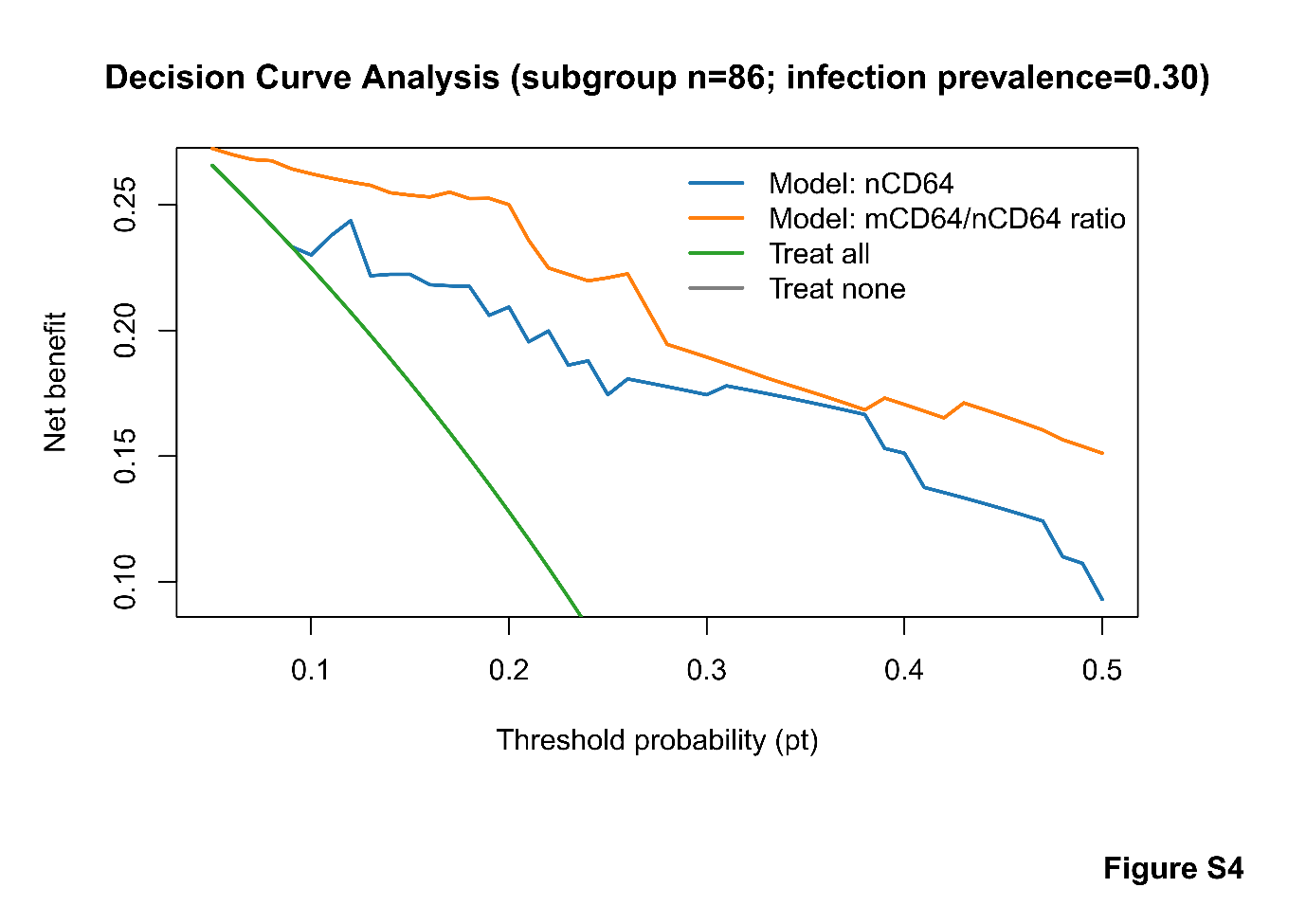


**Supplementary Figure S4. Decision curve analysis comparing neutrophil CD64 and the monocyte-to-neutrophil CD64 ratio in patients with systemic autoimmune and autoinflammatory diseases (n = 86).**

Decision curves show net benefit across a range of threshold probabilities (pt) for strategies based on neutrophil CD64 (nCD64) and the monocyte-to-neutrophil CD64 ratio (mCD64/nCD64 ratio), compared with treat-all and treat-none strategies. Threshold probability represents the minimum estimated probability of infection at which a clinician would adopt an infection-oriented management strategy, balancing the harms of unnecessary treatment against the harms of missed infection. Curves are displayed as point estimates without uncertainty bands to preserve interpretability given the limited sample size.


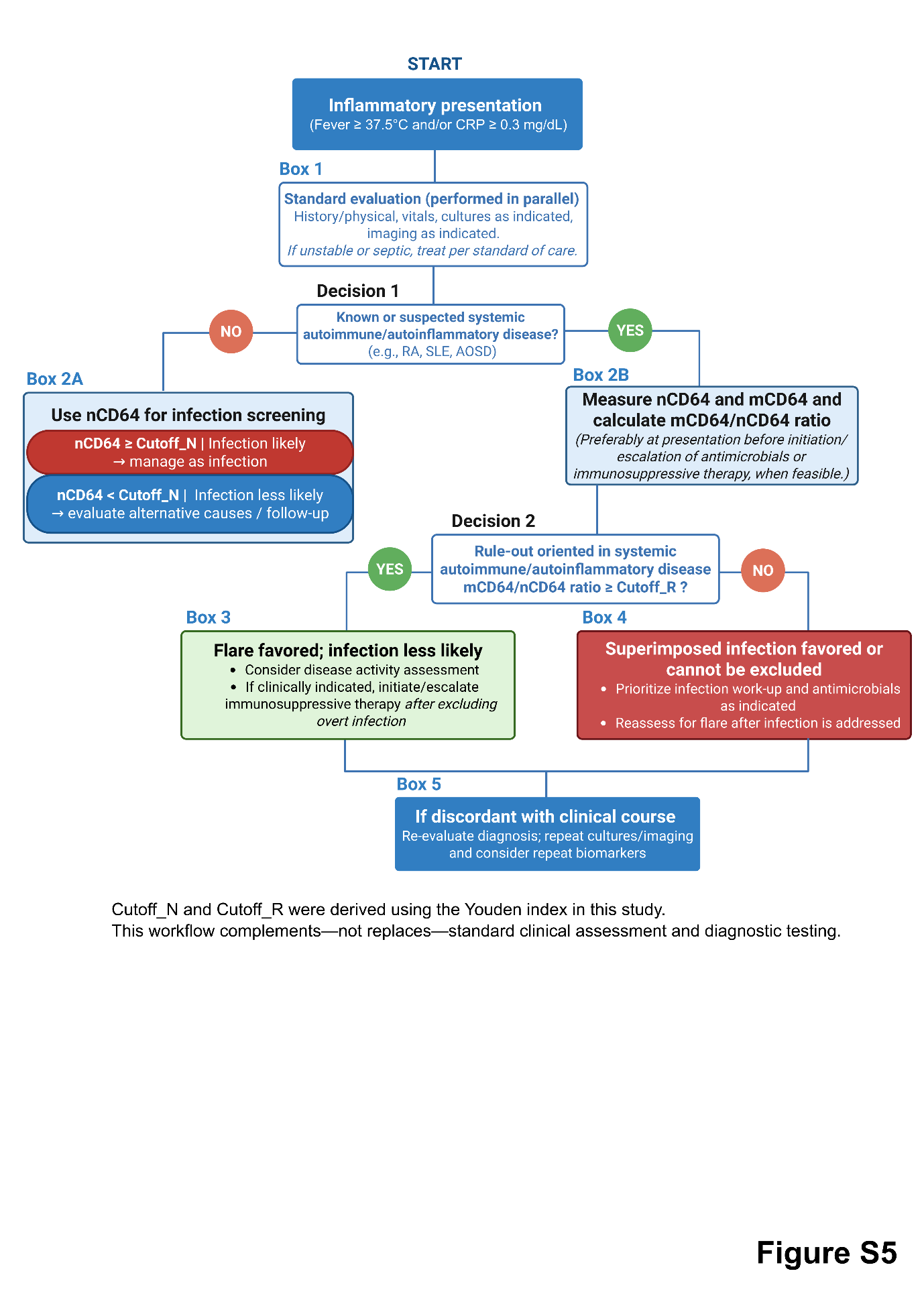


**Figure S5. This figure summarizes a stratified diagnostic approach proposed from our findings.** In unselected inflammatory presentations, nCD64 is positioned as a first-line biomarker for infection screening. In patients with systemic autoimmune and autoinflammatory diseases, where distinguishing flare from superimposed infection is clinically critical, the mCD64/nCD64 ratio is prioritized as an adjunct to support rule-out decision-making before initiating or escalating immunosuppressive therapy. Cutoff_N denotes the nCD64 threshold and Cutoff_R denotes the mCD64/nCD64 ratio threshold; both were derived using the Youden index. The figure was created using BioRender (<https://biorender.com/>).


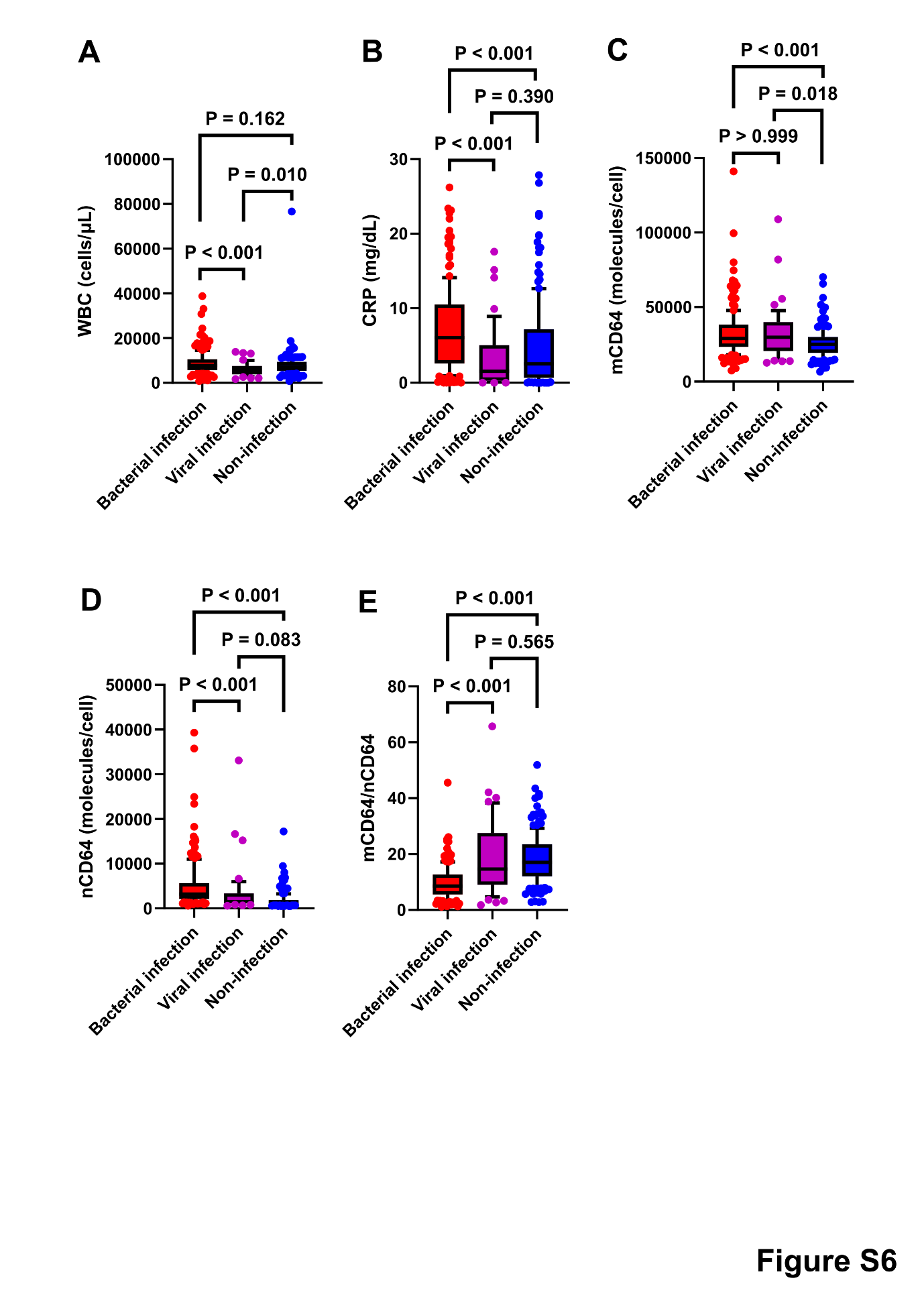


**Figure S6. Exploratory comparison of inflammatory biomarkers among bacterial infection, viral infection, and non-infectious inflammation in the overall cohort.**Box plots illustrating (A) white blood cell count (WBC), (B) C-reactive protein (CRP), (C) monocyte CD64 (mCD64), (D) neutrophil CD64 (nCD64), and (E) the mCD64/nCD64 ratio in patients with bacterial infection, viral infection, or non-infectious inflammation. Comparisons were performed using the Kruskal–Wallis test followed by Dunn’s multiple comparison test. nCD64 was significantly higher in bacterial infection than in viral infection or non-infectious inflammation, whereas no significant difference was observed between viral infection and non-infectious inflammation. In contrast, the mCD64/nCD64 ratio was significantly lower in bacterial infection than in viral infection or non-infectious inflammation, with no significant difference between viral infection and non-infectious inflammation. The middle line denotes the median, the box indicates the interquartile range, and the whiskers indicate the 10th–90th percentiles.


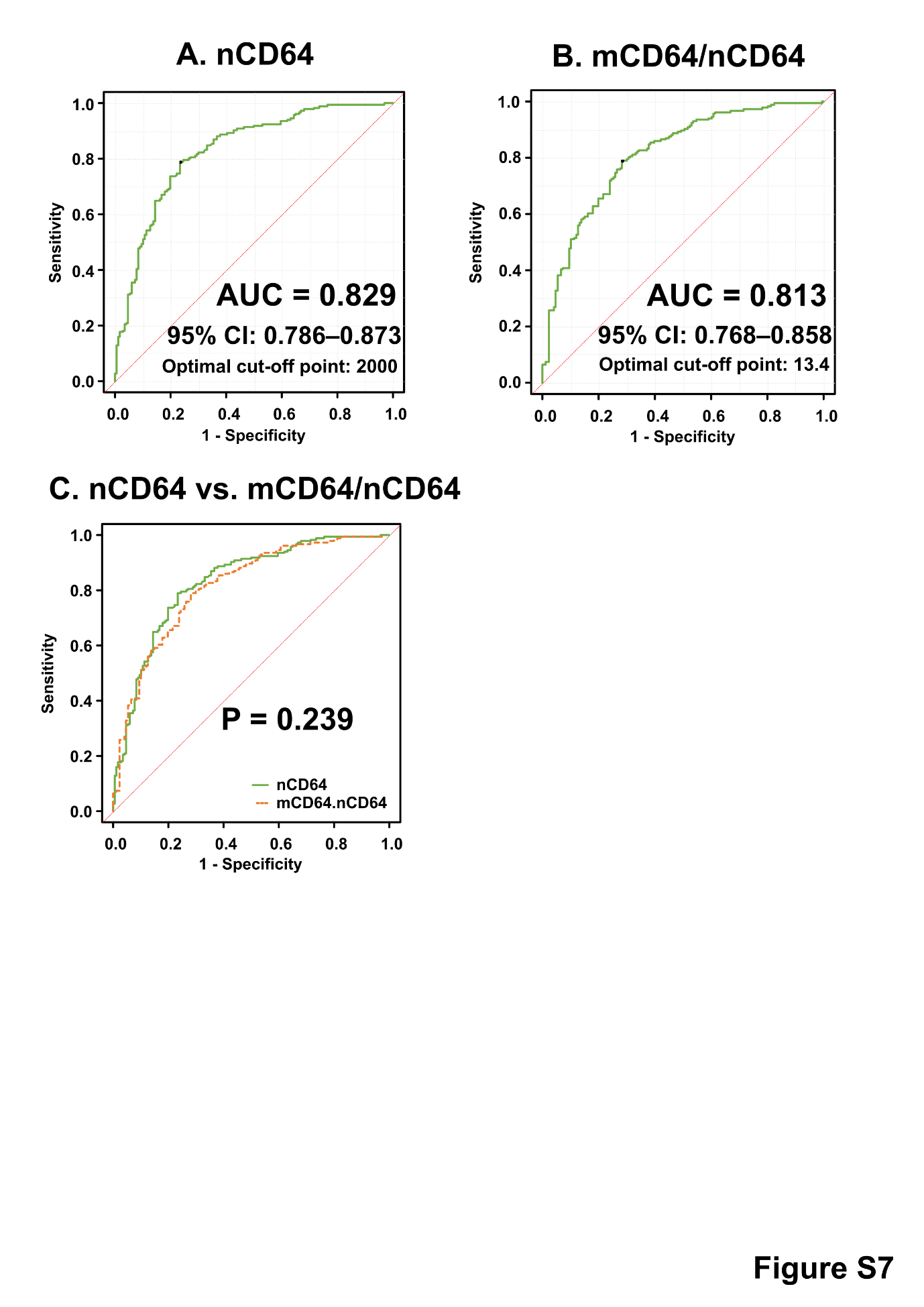


**Figure S7. Sensitivity analysis restricted to bacterial infection versus non-infectious inflammation.**Receiver operating characteristic (ROC) curves for (A) nCD64 and (B) the mCD64/nCD64 ratio in distinguishing bacterial infection from non-infectious inflammation after exclusion of viral infection, fungal infection, febrile neutropenia, and scabies. (C) Direct comparison of the ROC curves for nCD64 and the mCD64/nCD64 ratio. The AUC for nCD64 was 0.829 (95% CI 0.786–0.873), with an optimal cut-off point of 2000, sensitivity of 79.0%, and specificity of 76.5%. The AUC for the mCD64/nCD64 ratio was 0.813 (95% CI 0.768–0.858), with an optimal cut-off point of 13.4, sensitivity of 79.0%, and specificity of 71.7%. The difference between the two AUCs was not statistically significant (p = 0.239).

**Table S1.** Baseline characteristics of the subgroup of patients with systemic autoimmune and autoinflammatory diseases (n = 86)

| Variable | Infection Group (n = 26) | Disease Flare Group (n = 60) | p-value† |
| --- | --- | --- | --- |
| Demographics |  |  |  |
| Age, years, median (IQR) | 74 (61–82.8) | 70 (60.5–81) | 0.584 |
| Male sex, n (%)* | 4 (15.4) | 17 (28.8) | 0.276 |
| Medication |  |  |  |
| Glucocorticoid use, n (%) | 0 (0) | 8 (13.3) | 0.099 |
| Laboratory Findings |  |  |  |
| WBC, /μL, median (IQR) | 7,400 (5,775–10,000) | 6,250 (5,150–7,900) | 0.082 |
| CRP, mg/dL, median (IQR) | 3.7 (1.6–8.2) | 1.0 (0.3–4.1) | 0.010 |
| nCD64, molecules/cell, median (IQR) | 4,426 (2,304–8,259) | 1,318 (899–1,777) | < 0.001 |
| mCD64, molecules/cell, median (IQR) | 23,117 (18,570–31,047) | 22,883 (18,379–29,984) | 0.684 |
| mCD64/nCD64 Ratio, median (IQR) | 6.7 (3.5–9.2) | 16.7 (13.4–23.5) | < 0.001 |
| Underlying Diseases, n (%) |  |  |  |
| Rheumatoid arthritis | 20 (76.9) | 34 (56.7) | 0.092 |
| Adult-onset Still’s disease | 1 (3.8) | 3 (5.0) | > 0.999 |
| Systemic lupus erythematosus | 0 (0) | 2 (3.3) | 0.573 |
| Other diseases | 5 (19.2) | 21 (35.0) | 0.202 |
| Infectious Etiology & Focus, n (%) |  |  |  |
| Bacterial infection | 20 (76.9) | - | - |
| Respiratory tract | 13 (50.0) | - | - |
| Urinary tract | 2 (7.7) | - | - |
| Other bacterial foci (skin/soft tissue, abdominal, etc.) | 5 (19.2) | - | - |
| Viral infection | 3 (11.5) | - | - |
| Fungal infection | 2 (7.7) | - | - |
| Scabies | 1 (3.8) | - | - |

*Male sex unknown for 1 patient. †Mann–Whitney U test.

**Table S2.** Reclassification analysis comparing nCD64 alone and the mCD64/nCD64 ratio in the subgroup of patients without systemic autoimmune and autoinflammatory diseases (n = 322)

| Group | Total N | Improved by nCD64 (correct direction) | Worsened by nCD64 (wrong direction) | Net gain (NRI component) |
| --- | --- | --- | --- | --- |
| Events (Infection +) | 216 | 110 (50.9%) | 106 (49.1%) | 0.02 |
| Non-events (Infection -) | 106 | 76 (71.7%) | 30 (28.3%) | 0.43 |
| Total | 322 | - | - | **0.45 (p < 0.001)** |

Note: NRI, net reclassification improvement. The positive NRI value indicates that nCD64 alone provides superior classification compared to the ratio in this specific subgroup. “Improved” in non-events means that nCD64 correctly assigned a lower probability of infection than did the ratio.

**Text S1. Proposed stratified diagnostic approach using CD64 biomarkers**

**Overview and intent**

A visual summary of the proposed stratified diagnostic workflow is provided in Supplementary Figure S5. This supplementary text provides operational considerations for implementing and interpreting CD64 biomarkers in clinical practice. This approach is intended to complement—not replace—standard clinical assessment, microbiologic testing, and imaging, and it requires external validation before being used as a stand-alone decision tool.

**Clinical scenario**

Patient with fever and/or elevated inflammatory markers in whom infection versus non-infectious inflammation is being considered, including situations where clinicians must decide whether it is safe to initiate or escalate immunosuppressive therapy.

**1) Step 1: Define the clinical context (pretest probability)**

A. No known or suspected systemic autoimmune and autoinflammatory diseases

Proceed with general infection screening as outlined in Supplementary Figure S5 (Step 2A).

B. Known or suspected systemic autoimmune and autoinflammatory diseases (e.g., systemic lupus erythematosus, rheumatoid arthritis, adult-onset Still’s disease) where flare versus superimposed infection is the key dilemma

Proceed with the combined biomarker strategy as outlined in Supplementary Figure S5 (Step 2B).

**2) Specimen timing, handling, and basic quality considerations**

Timing of blood sampling

Whenever feasible, obtain blood at initial evaluation (i.e., at presentation) before initiation or escalation of antimicrobial or immunosuppressive therapy, because both infectious burden and treatment effects may influence biomarker levels.

If treatment has already been started, interpret results cautiously and prioritize the overall clinical trajectory.

Analytical considerations (flow cytometry–based CD64 quantification)

Use consistent pre-analytical handling (e.g., temperature-controlled storage) and analyze within the laboratory’s validated time window.

Ensure that gating strategy and calibration allow comparability across time points when longitudinal measurements are performed.

Febrile neutropenia / severe neutropenia (interpretation caveat)

nCD64-based interpretation requires a minimum number of neutrophil events for statistical reliability. If an adequate neutrophil gate cannot be obtained, avoid over-interpreting nCD64-derived indices and rely on the full clinical context.

**3) Interpretation principles (aligned with Supplementary Figure S5)**

Step 2A: General infection screening (no systemic autoimmune and autoinflammatory diseases)

Use neutrophil CD64 (nCD64) as the primary biomarker alongside standard evaluation.

In this context, nCD64 is expected to provide strong discrimination for infection, consistent with general-population performance.

Step 2B: Flare versus superimposed infection (systemic autoimmune and autoinflammatory diseases present)

In this context, measure both monocyte CD64 (mCD64) and nCD64, and calculate the mCD64/nCD64 ratio, recognizing that monocyte activation may be elevated in both flare and infection.

High ratio pattern (monocyte-dominant activation)

Interpreted as flare favored and infection less likely, particularly when clinical evaluation does not suggest a focal bacterial source.

Supports proceeding with disease activity assessment and, when clinically indicated, initiating or escalating immunosuppressive therapy after excluding overt infection.

Low ratio pattern, particularly when accompanied by high nCD64 (neutrophil-dominant activation)

Interpreted as superimposed bacterial infection favored or cannot be excluded.

Prioritize infection work-up and antimicrobial management as clinically indicated, and reassess inflammatory disease activity after infection is addressed.

Discordant scenarios (safety-net)

If biomarker patterns conflict with clinical course (e.g., persistent fever despite appropriate therapy, evolving imaging findings, or unexpected response patterns), reassess diagnosis, repeat cultures/imaging as needed, and consider repeat biomarkers at the clinician’s discretion.

**4) Cutoffs and generalizability**

Cutoff values for nCD64 and the mCD64/nCD64 ratio used in this study were data-derived (e.g., using ROC-based optimization such as the Youden index in the primary analysis framework) and should be regarded as study-specific rather than universal clinical thresholds.

The proposed workflow should be validated prospectively in larger, multi-center cohorts and across additional disease phenotypes and treatment settings (including varying immunosuppressive regimens) before broad adoption.
